# Understanding High-Functioning Depression in Adults

**DOI:** 10.7759/cureus.78891

**Published:** 2025-02-12

**Authors:** Judith F Joseph, Umit Tural, Nikeisha D Joseph, Teresa E Mendoza, Eshna Patel, Rachel Reifer, Margot Deregnaucourt

**Affiliations:** 1 Pediatric and Adult Psychiatry, Manhattan Behavioral Medicine, New York, USA; 2 Psychiatric Research, Nathan S. Kline Institute for Psychiatric Research, Orangeburg, USA

**Keywords:** depression, high-functioning, high-functioning depression, major depression, major depressive disorder

## Abstract

Introduction: High-functioning depression (HFD) is described as experiencing depressive symptoms such as fatigue, anhedonia, poor concentration, guilt, restlessness, sleep disturbances, and appetite changes without experiencing a lack of functioning or significant distress. The purpose of this study is to characterize the clinical correlates of HFD.

Methods: This study entailed a descriptive, cross-sectional design based on interviews administered to120 English-speaking participants with HFD (aged 18-75). The interview involved administering a semi-structured HFD Analysis Questionnaire, the Joseph HFD Inventory, the HFD Trauma Inventory, and the Joseph HFD Anhedonia Scale in a single, 30-minute session for each participant. Big traumas, defined as extremely traumatic events, were analyzed by the trauma inventory.

Results: Out of the 120 participants, 72 (60%) demonstrated HFD, and 17 (14%) demonstrated very HFD. A correlation was observed between symptoms of HFD, such as anhedonia and marital status, as post hoc tests showed that the average Anhedonia Scale score was higher for married or partnered participants than those who were single (p=0.038). As anticipated, the participants with higher Anhedonia Scale scores had higher HFD scores (p=0.003). These participants also experienced higher trauma inventory scores and big traumas. Furthermore, as participant education level increased, the number of big traumas reported decreased (p<0.001). Participants who were parents/caregivers of children also had the highest Anhedonia Scale and HFD scores (p=0.0126 and p=0.0210, respectively).

Conclusion: The results supported the hypothesis that individuals with HFD have increased levels of anhedonia and trauma. However, trauma scores were inversely associated with education level in HFD.

## Introduction

Depression is one of the most disabling mental health disorders among the leading causes of global health-related burdens [1]. In 2019, an estimated 7.8% of adults in the United States (US) experienced an episode of major depressive disorder (MDD), and 5.3% reported that they had a depressive episode that led to significant impairment in that same year [2]. According to the Diagnostic and Statistical Manual of Mental Disorders, Fifth Edition (DSM-5), to meet the criteria for MDD, an individual must exhibit depressed mood or lack of enjoyment along with five or more additional criteria (e.g., weight changes, insomnia, worthlessness, suicidal thoughts) for most of the day for two weeks or longer [3]. Similarly, the DSM-5 explains that to meet the criteria for persistent depressive disorder (PDD), a person must display a depressed mood for most of the day and two or more additional criteria (e.g., poor appetite, overeating, hopelessness, insomnia) almost daily for two years or longer [3].

In many cases, symptoms of depression may not be acknowledged until burnout is reached, with research showing that physician burnout is becoming an increasing problem in the US [4,5]. However, although individuals with MDD typically exhibit long-term symptoms that may gradually become debilitating, people with what is termed high-functioning depression (HFD) are difficult to assess and treat because they do not readily identify with being depressed. In addition, people with HFD may learn to cope with their symptoms by becoming high-functioning to the point where they distract themselves from negative emotions or a lack of feeling emotions. Such individuals may use work or daily activities (e.g., caregiving, errand-completing) to distract themselves from acknowledging persistent signs of HFD, such as negative emotions and anhedonia. This could also help to explain why most people with suspected HFD do not recognize the signs of depression, even though some of them report feeling exhausted or numb. Additionally, because individuals with HFD are high-functioning, they are not generally diagnosed with MDD in clinical settings because they do not exhibit impairment or a lack of functioning, nor do they seem to be in distress.

It is important to note that the term "high-functioning depression" is not an official diagnosis according to mental health manuals such as the DSM-5. However, the concept has gained popularity in society and among mental health professionals to describe people who meet the symptoms of MDD but who can maintain a level of functionality in their daily lives. This concept is a combination of various approaches in psychology and psychiatry that recognize that depression can manifest itself at different levels of severity and does not always result in a total loss of functionality. The important point to remember is that although HFD is not an official diagnosis, it is still a serious form of depression that can significantly affect a person's quality of life. Recognizing and treating these symptoms can help improve emotional and overall well-being.

It is hypothesized that individuals with HFD learn to cope with symptoms, such as anhedonia, a lack of emotion, sadness, or distress, by distracting themselves from negative emotions or a lack of feeling emotions through their lifestyle and career choices, relationships (e.g., spousal or partner interactions), or caregiving activities (e.g., caring for children or relatives). Thus, they become high-functioning while experiencing substantial levels of depression. Furthermore, individuals with HFD appear to employ a form of psychological resilience through multisystemic dynamic processes that function as defense mechanisms against the long-term impact of intense daily stress or trauma [6]. Another potential coping or defense mechanism involves sublimation, in which individuals may channel unacceptable urges, such as quitting a job or giving up, into a productive outlet. Sublimation is described as both a redirection of energy and a defense mechanism to help reduce intrapsychic conflict [7,8] (e.g., resigning from a strenuous job or resting from caregiving). This type of redirection may lead to HFD.

This high-functioning ability makes it hard for individuals to recognize the signs of HFD or motivate them to seek treatment. Similarly, in clinical practice, it is often challenging to identify and treat individuals with HFD who have found ways to cope with their symptoms through various mechanisms, including taxing daily life activities. Over time, however, the symptoms associated with HFD may gradually worsen, and the ability to employ resilience or sublimation may also weaken. This may gradually lead to burnout, impairment, or a lack of functioning, which is unmistakable, as seen in classical depression.

HFD is distinct from burnout, which is described as an occupational disorder that is characterized by mental, physical, and emotional exhaustion due to prolonged stress in a working environment [5]. Factors associated with burnout include a high workload, poor career-life balance, and loss of autonomy, among others [9-11]. Clinicians who experience burnout often have a higher incidence of medical errors and absenteeism, as well as lower patient satisfaction rates [12,13]. Conversely, clinicians with HFD, a mood disorder, typically maintain their ability to function efficiently and sustain a sufficient career-life balance, although they may report feeling drained or numb at times. Burnout, on the other hand, would result in noticeable impairments in daily and work-related tasks, while these types of impairments are not typically experienced with HFD. Unlike people with burnout, individuals with HFD may display “smiling depression,” where they are able to continue completing their daily tasks and upholding important responsibilities while experiencing mental health issues.

Similarly, HFD is different from dysthymic disorder and PDD, which are chronic mood disorders labeled as mental illnesses with a significant disease burden and have symptoms that are often more debilitating than episodic major depression [14,15]. These two disorders are less severe than MDD but last longer. People diagnosed with either of these conditions often report feeling depressed for prolonged periods or feeling as if they have recurring bouts of persistent depression [14,15]. This is distinct from HFD, where individuals do not usually report feeling depressed, as they have learned to distract themselves from negative emotions or feelings that would indicate depression and still function efficiently in home and work settings. That is, people with HFD are able to perform at work and maintain healthy, normal relationships, despite experiencing mild depression.

In addition, to be diagnosed with PDD and/or MDD, an individual has to meet criteria that demonstrate their symptoms cause significant distress and impaired functioning. HFD is different from these disorders because people do not report substantial distress and display high functioning or exceed functioning expectations on a daily basis. Similarly, symptoms that indicate burnout include depleted energy levels, weakness, persistent unhappiness, negative attitudes, and the loss of the ability to work effectively or maintain relationships. These types of symptoms are not typically experienced or reported by those suffering from HFD. Thus, HFD is clearly different from PDD, MDD, and burnout and warrants further exploration.

Currently, as HFD is not recognized in the DSM-5, this limits access to mental health care for people who may be unsuspectingly dealing with this issue. Insurance companies do not cover mental health services unless a health issue is designated with an International Classification of Diseases (ICD 10) or current procedural terminology (CPT) code. Therefore, an individual presenting with symptoms, including feeling drained or numb, who may be displaying additional signs of HFD, such as anhedonia and negative emotions, may not be referred for treatment until their symptoms reach the DSM-5 criteria for impairment or significant distress. Similarly, some individuals may not seek treatment until they are severely impaired or in emotional crisis. Other individuals with HFD may have low rates of treatment because their symptoms go undetected by themselves and their healthcare providers. However, depression is a mood disorder, and if HFD symptoms are left untreated, like typical depression, the issues can become persistent, intensify, and develop into clinical depression, also known as MDD [16], which is one of the leading causes of disability [17,18].

The purpose of this interview study is to help define, describe, and characterize the signs and symptoms of HFD in an effort to develop approaches that successfully treat this issue. An additional aim involves identifying risk factors for developing HFD to identify individuals who may have a high susceptibility to this health issue. It is hypothesized that people with HFD are often underdiagnosed because they do not display significant impairments in daily functioning and because they do not acknowledge or report negative emotions as readily as people with typical depression. Instead, what distinguishes people with HFD from those with typical presentations of depression is that individuals with HFD appear to function at high levels of efficiency. It is also proposed that HFD is characterized by high levels of anhedonia and is associated with past trauma, despite an individual’s high level of functioning.

Therefore, we designed an interview study that aims to identify this underdiagnosed and undertreated form of depression to determine the clinical correlates of HFD, including associations with traumatic experiences and characteristics of individuals who may be susceptible to HFD. The primary and secondary outcomes include assessing the presence of distinctive characteristics of HFD, assessing whether trauma occurs at higher rates for people with HFD, and assessing demographics, lifestyle patterns, and risk factors associated with HFD. By reviewing widely administered psychometric questionnaires regarding anhedonia, adverse childhood events, and depression, such as the Snaith-Hamilton Pleasure Scale (SHAPS), Adverse Childhood Experiences (ACEs), and the DSM-5, we developed several clinician-administered HFD inventories that were administered during a single interview to assess risk factors and symptoms of HFD. It is anticipated that certain demographics may be more susceptible to HFD due to their line of work, marital or familial status, cultural differences, and other factors. Shifting the focus to effective treatment for people with HFD through improved assessment and diagnostic tools can help lower the burden of mental health epidemics associated with chronic depressive issues.

## Materials and methods

Participants and study design

This study entailed a population-based, descriptive, cross-sectional design based on interviews from (October 2023) through (ongoing), administered to 120 English-speaking participants with HFD (aged 18-75) who did not meet the criteria for major depression. Participants in this study were patients in the Manhattan Behavioral Medicine database who identified as having HFD and requested to participate. Interviews were conducted at the Manhattan Lab of Manhattan Behavioral Medicine, although participants were from all over the US. Eligible participants met the following criteria: 1) able to speak, read, and comprehend English (e.g., at least with moderate to good conversational proficiency); 2) willing to provide verbal and written consent to participate in the interview; 3) aged 18 and over; and 4) presented with symptoms of depression but were experiencing HFD. As this was a pilot study, most participants were single females with higher education degrees (e.g., college graduate, graduate degree) and were recruited from the Manhattan Behavioral Medicine database or through site recruitment. All participants were screened using the HFD Analysis Questionnaire, which included the HFD Trauma Inventory (HFD-TI). Assessments using the Joseph HFD Inventory (HFD-I) and the Joseph HFD Anhedonia Scale (HFD-AS) were performed for participants with HFD by an on-site clinician.

This population-based, descriptive clinical research report was exempted by the Advarra Institutional Review Board (IRB). Using the Department of Health and Human Services regulations found at 45 CFR 46.104(d)(2), the Advarra IRB determined that this research project is exempt from IRB oversight. The Advarra IRB also completed the necessary additional limited review considerations as set forth under the Revised Common Rule, 45 CFR 46.104(d). At the completion of the Advarra IRB review, all study-related documents were removed from the active files and archived. The Advarra IRB granted this exemption with an understanding of the following:

1. The research project would only be conducted as submitted and presented to the IRB, without additional change in design or scope.

2. Should the nature of the research project, or any aspect of the study, change such that the nature of the study no longer meets the criteria found in 45 CFR 46.104(d)(2), the lead researcher will resubmit revised materials for IRB review.

3. It is the responsibility of each investigator to ensure that the project meets the ethical standards of the institution. Specifically, the selection of subjects is equitable, there are adequate provisions to maintain the confidentiality of any identifiable data collected, and when there are interactions with research subjects, they will be informed of the following: that the activity involves research, a description of the procedures, that participation is voluntary, and the contact information for the researcher.

Following the Advarra IRB review, written informed consent was obtained from all participants. The 30-minute interview entailing the administration of the semi-structured HFD Analysis Questionnaire, the Joseph HFD-I, the HFD-TI, and the Joseph HFD-AS was administered in a single session for each participant, and no medical procedures or treatments were administered. All participant details were de-identified. The interview study did not involve the administration of any drug, medical treatment, or therapeutic procedure. It was anticipated that the participants would continue with any treatments prescribed by their treating physician. No adverse events were expected as a result of the interview, as no type of drug, medical procedures, or treatments were administered.

Data collection

A semi-structured HFD Analysis Questionnaire was used to assess the socio-demographic characteristics, behavioral risk factors, and the current level of functioning, sadness, and distress during an HFD episode. The semi-structured questionnaire consisted of items that assessed age, gender, highest education level, occupation, marital status, level of functioning, and treatment history for the characterization of the population.

For the assessment of HFD, the Joseph HFD-I was administered to the participants. Clinicians at Manhattan Behavioral Medicine constructed this inventory to measure symptoms observed in clinical practice that are linked to HFD, such as anhedonia, fatigue, periods of sadness, empty mood, and lack of emotion. The inventory comprises 12 questions (yes or no answers) intended to help define signs and symptoms that characterize HFD. Widely administered psychometric questionnaires regarding anhedonia, adverse childhood events, and depression, such as SHAPS, ACEs, and the DSM-5, were used as guides to create this inventory that measures HFD.

For the assessment of trauma, the DSM-5 was used to create the HFD-TI that was administered to participants. Limited access to validated questionnaires that measure HFD warranted the development of this inventory, as the instruments that are currently available (e.g., SHAPS, ACES, and the Philadelphia Expanded ACE Survey) are antiquated, dated, and not applicable to the targeted population of individuals with suspected HFD. Thus, relevant questionnaires were designed based on previously developed instruments to evaluate the presence of HFD in individuals identified as high-functioning. This inventory groups trauma into four types: childhood (before the age of 18), intergenerational, mass, and adult lifespan. Administration of the interview involves asking participants to identify traumatic events they experienced at different stages of their lives to group them into one of the four groups of traumas. This clinician-administered tool comprises 46 questions (yes or no answers) that reflect traumatic experiences across the four types of trauma, such as witnessing extreme violence during childhood (e.g., a stabbing or shooting), having an abusive or suicidal partner during adulthood, having relatives who survived genocide (intergenerational trauma), and surviving a war or major disaster (mass trauma). Within each trauma category are several items that indicate the occurrence of a big trauma (big T) or an extremely traumatic event, such as experiencing physical abuse with scarring during childhood, being sexually assaulted during adulthood, having immediate relatives who were refugees (intergenerational trauma), or surviving a major fatal disaster like an earthquake or flood (mass trauma). Designating specific inventory items as indicators of big trauma provided an assessment of the impact of extreme trauma on participants with HFD.

Widely used questionnaires, such as the ACES and the Philadelphia Expanded ACE Survey developed by Cronholm et al., 2015 [19], which measure childhood exposure to trauma, and the original Centers for Disease Control and Prevention (CDC) Kaiser Permanente Study, along with experiences derived from clinical practice, were used to construct the HFD-TI. The CDC Kaiser Permanente Study was one of the largest investigations of the relationship between childhood neglect, abuse, and family or household challenges and later-life health and behaviors [20].

For the assessment of anhedonia, the Joseph HFD-AS was administered. This clinician-administered scale comprises 18 statements (agree or disagree responses) that measure the presence and severity of anhedonia, which, along with trauma, is linked to HFD. The most commonly used scale for anhedonia is the SHAPS, which is frequently used in clinical research. The items that comprise SHAPS are dated, antiquated, and not applicable to the targeted population of individuals with HFD seen in the clinic. Therefore, clinicians at Manhattan Behavioral Medicine adopted a new anhedonia scale based on SHAPS developed by Snaith et al., 1995 [21].

The interview entailing the administration of the semi-structured HFD Analysis Questionnaire, the Joseph HFD-I, the HFD-TI, and the Joseph HFD-AS was administered in a single session lasting approximately 30 minutes for each participant. For the HFD-I, the Trauma Inventory, and the Anhedonia Scale, an answer of "Yes" was accepted as 1, and "No" was accepted as 0. Similarly, an answer of "Agree" was accepted as 1, and "Disagree" was accepted as 0. The sum of all answers in the instruments generated the total score of the inventory or scale. No cut-off score or interpretation level (i.e., low or high) was described. The results presented are based on the numerical, scalar, and quantitative differences or comparisons.

Statistical analyses

Demographics were reported as means, standard deviations (SD), and frequencies, and elements of descriptive and bivariate statistics were used. Means and SD, as well as medians and interquartile ranges (IQRs), were used to summarize the characteristics of the variables. Total scores of the HFD, trauma, and anhedonia scales and the number of big traumas were reported as mean ± SD and median ± IQR, depending on the distributional features of the variable. The chi-square test was used to compare frequency distributions of categorical variables, and the comparison of means and medians was performed by using Student’s t-tests or Mann-Whitney U-tests, depending on the distributional features of data as assessed by the Shapiro-Wilk test. The Kruskal-Wallis test was used to compare three or more groups, and the Jonckheere-Terpstra (JT) linear trend test or Wilcoxon rank sum tests were used to follow up a significant Kruskal-Wallis test as post hoc tests. The Spearman or Pearson correlation tests were used to evaluate the correlations between the psychometric scales, depending on the distributional characteristics of the data. The linear-by-linear chi-square test was used to evaluate directional associations between ordinal variables. Statistical analyses were performed in R software (R Foundation, Vienna, Austria), and a p-value of less than 0.05 was used to denote statistical significance. The Bonferroni p-value adjustment method was used for multiple testing.

## Results

Demographics

A total of 120 participants were included in the analysis (female: n=77, male: n=35, non-binary: n=8). The mean age of participants was 40.87±14.36; 40% were single, and 45% were college graduates. The details of the participants' demographic features are summarized in Table 1.

**Table 1:** Demographics of the sample

	Total sample (N=120)	Females (N=77)	Males (N=35)	Non-binary (N=8)	Statistical test
Marital status	Single (N=48, 42.1%)	25 (34.7%)	17 (50%)	6 (75.0%)	χ^2^=8.413, df=6, p=0.2094
	Married (N=46, 40.4%)	34 (47.2%)	11 (32.4%)	1 (12.5%)	
	Divorced/separated/widowed (N=17, 14.9%)	10 (13.9%)	6 (17.6%)	1 (12.5%)	
	Not provided (N=3, 2.6%)	3 (4.2%)	0 (0%)	0 (0%)	
Race	White (N=78)	49 (63.6%)	23 (65.7%)	6 (75.0%)	χ^2^=3.7454, df=8, p=0.8793
	African American (N=20, 16.7%)	14 (18.2%)	6 (17.1%)	0 (0%)	
	Asian (N=9, 7.5%)	5 (6.5%)	3 (8.6%)	1 (12.5%)	
	Other (N=7, 5.8%)	4 (5.2%)	2 (5.7%)	1 (12.5%)	
	Not provided (N=6, 5.0%)	5 (6.5%)	1 (2.9%)	0 (0%)	
Ethnicity	Non-Hispanic (N=86, 72.9%)	58 (75.3%)	24 (70.6%)	4 (57.1%)	χ^2^=2.2171, df=4, p=0.6959
	Hispanic (N=20, 16.9%)	13 (16.9%)	5 (14.7%)	2 (28.6%)	
	Not provided (N=12, 10.2%)	6 (7.8%)	5 (14.7%)	1 (14.3%)	
Highest education level	High school graduate (N=13, 10.8%)	6 (7.8%)	5 (14.3%)	2 (25.0%)	χ^2^=4.3705, df=6, p=0.6267
	Some college (N=24, 20.0%)	18 (23.4%)	5 (14.3%)	1 (12.5%)	
	College graduate (N=54, 45.0%)	36 (46.8%)	15 (42.9%)	3 (37.5%)	
	Graduate degree (N=29, 24.2%)	17 (22.1%)	10 (28.6%)	2 (25.0%)	
Caretaker	Caretaker of parents/elderly relative (N=12, 10.5%)	8 (11.1%)	4 (11.8%)	0 (0%)	χ^2^=1.5154, df=4, p=0.8239
	Neither (N=80, 70.2%)	50 (69.4%)	23 (67.6%)	7 (87.5%)	
	Parent/caretaker of children (N=22, 19.3%)	14 (19.4%)	7 (20.6%)	1 (12.5%)	

High-functioning depression analysis of distress, sadness, and level of functioning

Out of the 120 participants, 72 (60%) were classified as high-functioning and 17 participants (14%) were classified as very high-functioning. Forty-seven participants (39.2%) were rated as moderately distressed, and levels of distress were significantly associated with the Joseph HFD-AS total scores and Joseph HFD-I total scores. Similarly, levels of sadness were significantly associated with the HFD-AS total and DI total scores. However, the level of functioning was not significantly associated with the HFD-AS, HFD-I, or HFD-TI total scores.

Gender identity, age, and psychometric scale scores

For gender identity, the means, median values, and statistical test results of the continuous variables, including age and psychometric evaluations, can be seen in Table 2.

**Table 2:** Means and medians of psychometric assessments by gender identity HFD-AS: High-Functioning Depression Anhedonia Scale; HFD-I total: High-Functioning Depression Inventory total; HFD-TI total: High-Functioning Depression Trauma Inventory total; q1, q3: first and third quartiles; IQR: interquartile range; MAD: mean absolute deviation; SD: standard deviation; SE: standard error; CI: confidence interval

Gender identity	Variable	n	Min	max	Median	q1	q3	IQR	MAD	Mean	SD	SE	CI	Statistics
Total population	Age	119	18	75	38	30.5	52	21.5	17.791	40.866	14.357	1.316	2.606	NA
Female	Age	76	18	75	44.5	30.25	53	22.75	15.567	41.816	14.419	1.654	3.295	Kruskal–Wallis (χ^2^)=10.078, df=2, p=0.0065
Male	Age	35	18	75	37	32	50	18	13.343	42.114	14.094	2.382	4.841	
Non-binary	Age	8	18	32	25.5	23.75	31.25	7.5	5.93	26.375	4.984	1.762	4.167	
Total population	HFD-AS total	116	0	18	11	8	13	5	4.448	10.733	4.078	0.379	0.75	NA
Female	HFD-AS total	74	0	18	12	8.25	14.75	6.5	4.448	11.297	4.03	0.468	0.934	Kruskal–Wallis (χ^2^)=3.931, df=2, p=0.1401
Male	HFD-AS total	34	1	17	9	7	13	6	4.448	9.588	4.321	0.741	1.508	
Non-binary	HFD-AS total	8	6	13	11.5	8.75	12	3.25	1.483	10.375	2.446	0.865	2.045	
Total population	HFD-I total	119	2	12	11	9	11	2	1.483	10.185	1.799	0.165	0.327	NA
Female	HFD-I total	77	2	12	11	9	11	2	1.483	10.156	1.686	0.192	0.383	Kruskal–Wallis (χ^2^)=0.948, df=2, p=0.6224
Male	HFD-I total	34	4	12	11	9	12	3	1.483	10.206	2.129	0.365	0.743	
Non-binary	HFD-I total	8	8	12	11	9	11.25	2.25	1.483	10.375	1.506	0.532	1.259	
Total population	HFD-TI total	120	0	37	14	9	20.25	11.25	8.896	14.808	7.391	0.675	1.336	NA
Female	HFD-TI total	77	0	37	13	8	20	12	8.896	14.299	7.804	0.889	1.771	Kruskal–Wallis (χ^2^)=4.312, df=2, p=0.1158
Male	HFD-TI total	35	5	30	14	10.5	20	9.5	7.413	14.971	6.675	1.128	2.293	
Non-binary	HFD-TI total	8	12	25	19.5	15.25	23.25	8	5.93	19	5.292	1.871	4.424	
Total population	Big trauma	120	0	14	4	2	7	5	2.965	4.75	3.101	0.283	0.561	NA
Female	Big trauma	77	0	14	4	2	7	5	2.965	4.494	3.169	0.361	0.719	Kruskal–Wallis (χ^2^)=4.957, df=2, p=0.0839
Male	Big trauma	35	0	11	5	3	7	4	2.965	4.857	2.952	0.499	1.014	
Non-binary	Big trauma	8	3	10	7	4.75	9	4.25	2.965	6.75	2.605	0.921	2.178	

The median values for HFD-AS total, HFD-I total, and HFD-TI total scores and the number of big traumas were not significantly different among gender identities. However, the median age (25.5) was significantly lower in the non-binary group than in males (37) and females (44.5) (Kruskal-Wallis (ꭓ^2^)=10.08, p=0.007) (Table 2). The median age was not significantly different between males and females. Age was significantly and positively correlated with HFD-AS scores (ρ=0.202, p=0.030). However, for the total population, the means of the HFD-AS total, the HFD-I total, and the HFD-TI total scores were 10.7±4.08, 10.2±1.80, and 14.8±7.39, respectively. There were no significant differences in psychometric scale scores and gender identity (Table 2).

Impact of education level on trauma experience and psychometric scale scores

**Figure 1:**
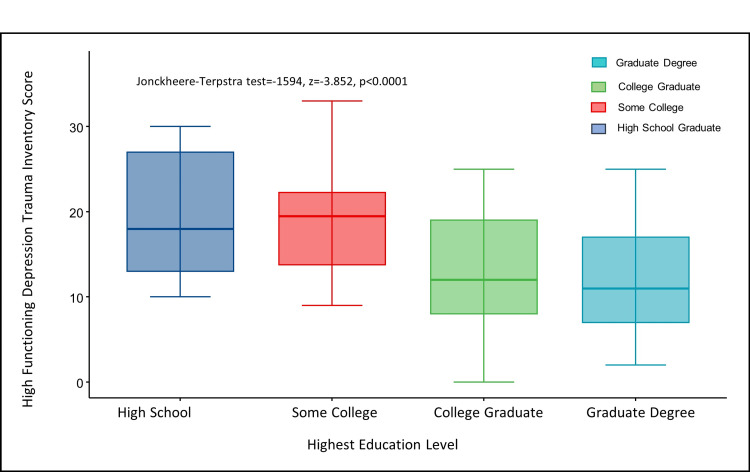
HFD-TI scores decreased as education level increased HFD-TI: High-Functioning Depression Trauma Inventory

For the psychometric scale scores, the medians of the HFD-AS scores and the HFD-I scores were not significantly different across education levels. However, the HFD-TI scores were significantly different across education levels, as participants with graduate-level education had significantly lower HFD-TI scores than other educational levels (Figure 1). The JT trend test also demonstrated a significant linear decreasing trend in HFD-TI scores as education level increased (Figure 1).

**Figure 2:**
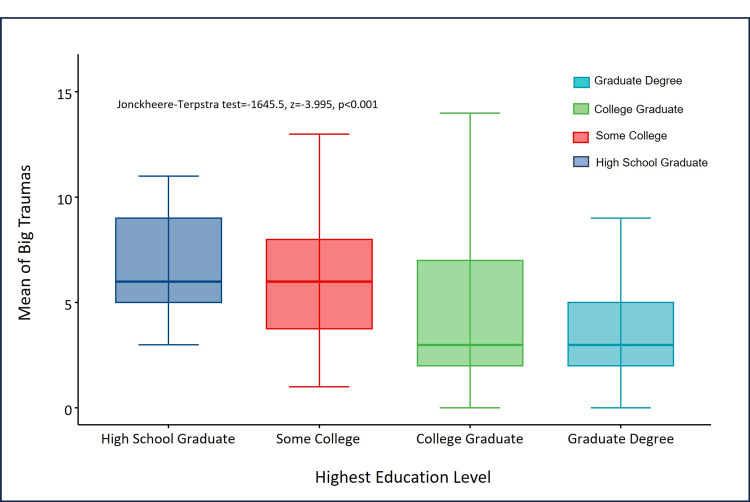
Decreasing trend in the number of big traumas with increasing education levels

The median number of big traumas was not significantly different between gender identity groups (Kruskal-Wallis (χ^2^)=4.9567, df=2, p=0.08388). However, the median number of big traumas was significantly different in education level groups (Kruskal-Wallis (χ^2^)=17.705, df=3, p=0.000506) and marital status groups (Kruskal-Wallis (χ^2^)=7.1612, df=2, p=0.02786). The JT trend test showed a significant linear decreasing trend in the mean number of big traumas as education level increased (JT=1645.5, z=-3.995, p<0.0001) (Figure 2).

Correlations among demographics, psychometric scale scores, and big traumas

**Figure 3:**
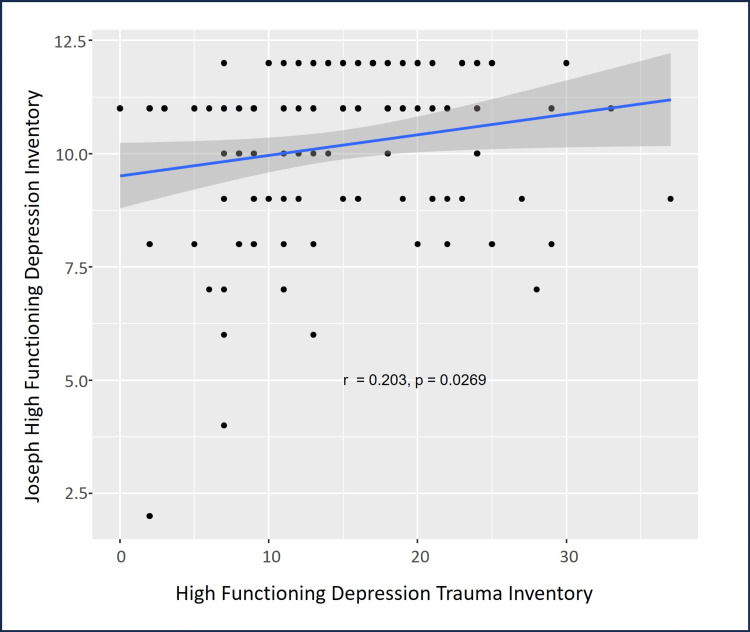
Psychometric scale scores showing a positive correlation between HFD and trauma. The blue line indicates the regression line with 95% confidence intervals indicated by the dark gray shaded area HFD: high-functioning depression

**Figure 4:**
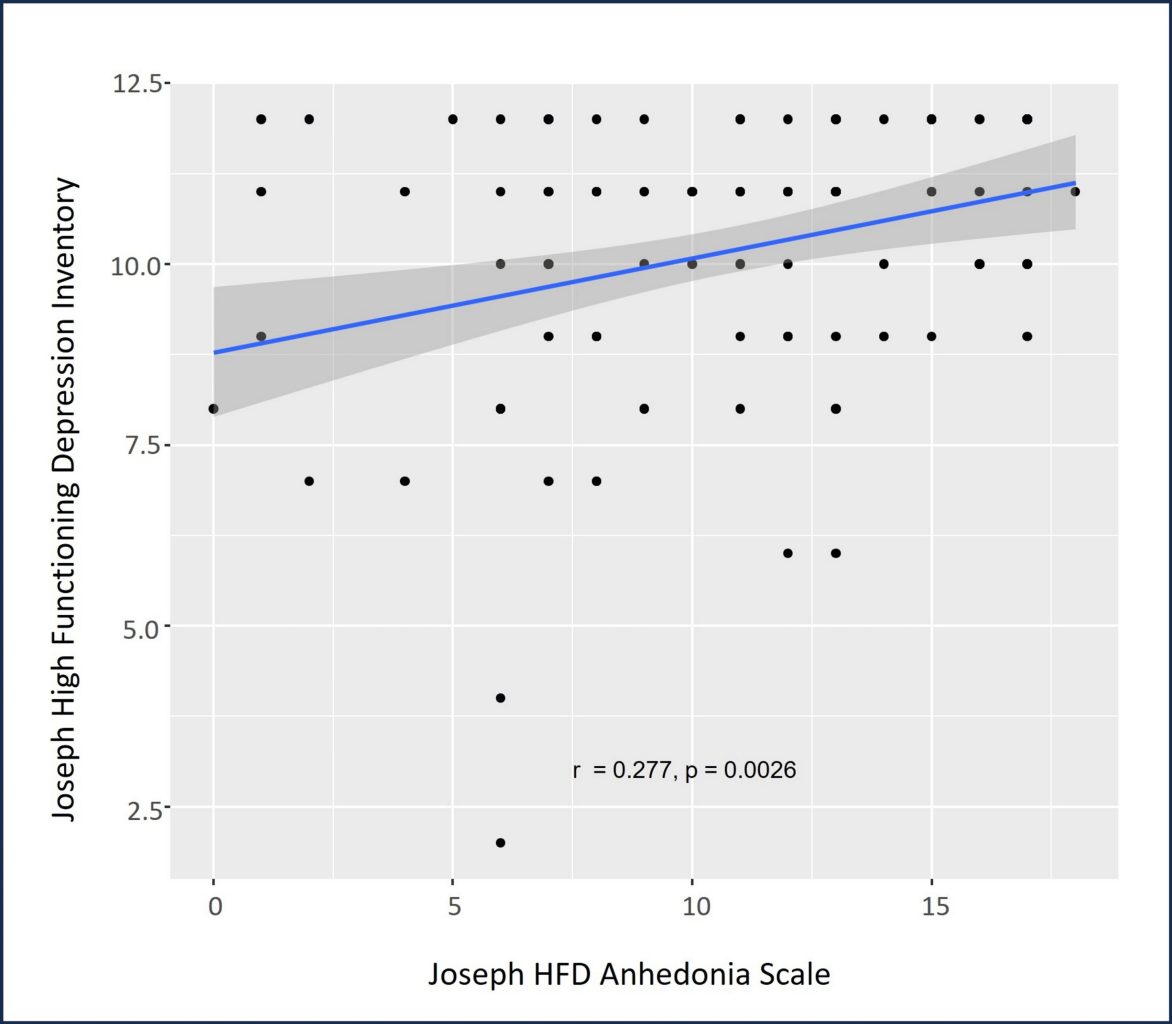
Psychometric scale scores showing a positive correlation between anhedonia and HFD. The blue line indicates the regression line with 95% confidence intervals indicated by the dark gray shaded area HFD: high-functioning depression

**Figure 5:**
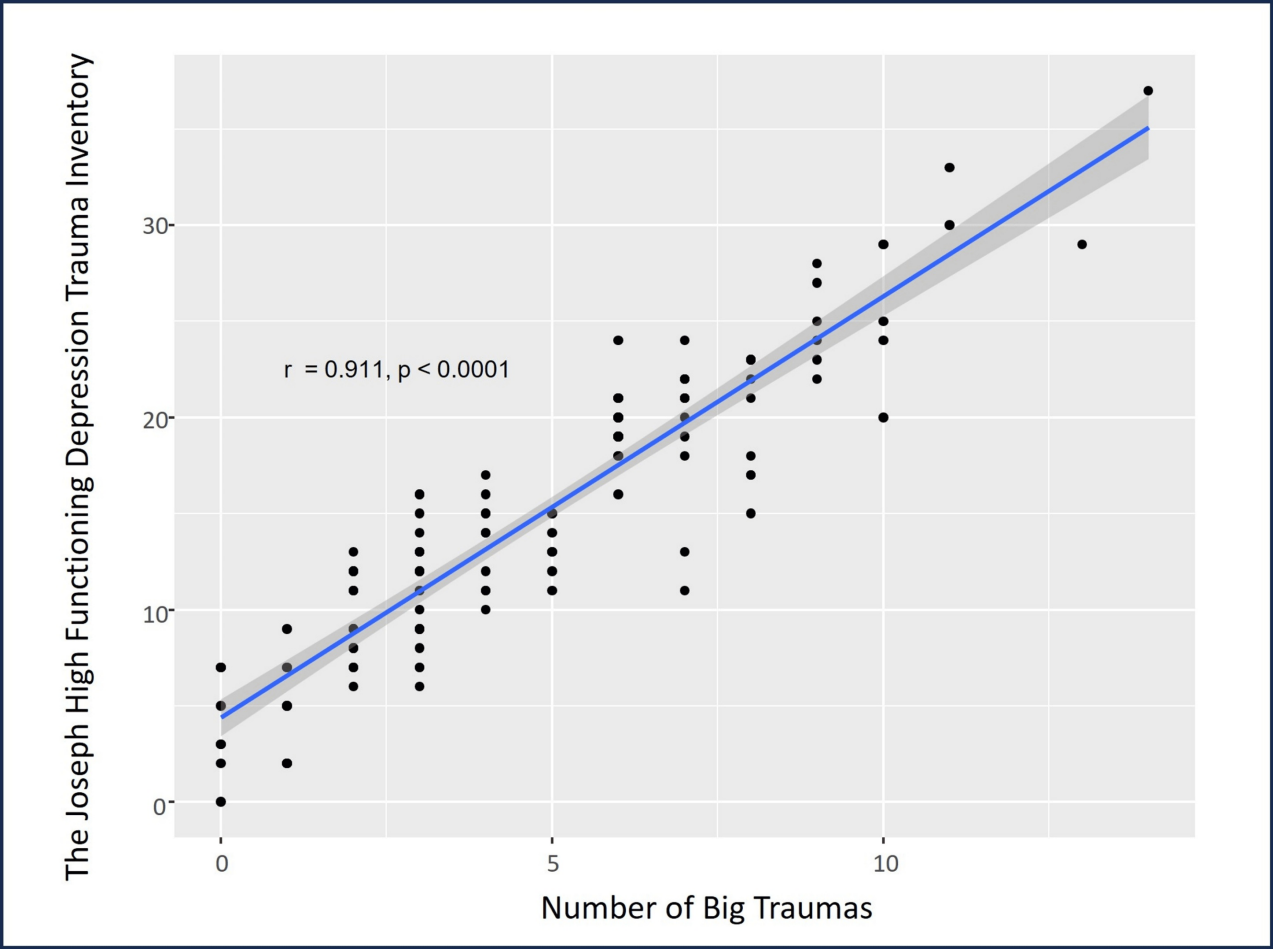
Correlation analyses between the HFD-TI scores and big trauma demonstrating a strong positive correlation. The blue line indicates the regression line with 95% confidence intervals indicated by the dark gray shaded area HFD-TI: High-Functioning Depression Trauma Inventory

There were statistically significant correlations among the psychometric assessment tools’ scores. HFD-I scores were significantly and positively correlated with HFD-TI scores (ρ=0.203, p=0.027, Figure 3), and HFD-AS scores were significantly and positively correlated with HFD-I scores (ρ=0.277, p=0.003, Figure 4). Additionally, age was significantly and positively correlated with HFD-AS scores (ρ=0.202, p=0.030). As anticipated, the number of big traumas was positively and significantly correlated with HFD-TI scores (ρ=0.911, p<0.0001, Figure 5).

The Kruskal-Wallis test showed a significant difference in the number of traumas among singles, married participants, and widowed participants (Kruskal-Wallis (χ^2^)=7.1612, df=2, p=0.02786). In particular, post hoc tests showed that the median big trauma score was significantly lower in the married or partnered group compared to the single group (Bonferroni-adjusted, p=0.029). HFD-AS scores were significantly different in marital status groups (Kruskal-Wallis (χ^2^)=6.3303, df=2, p=0.04221). The post hoc test showed that the median HFD-AS score was higher in the married or partnered group than for those who were single (Bonferroni-adjusted, p=0.038).

**Figure 6:**
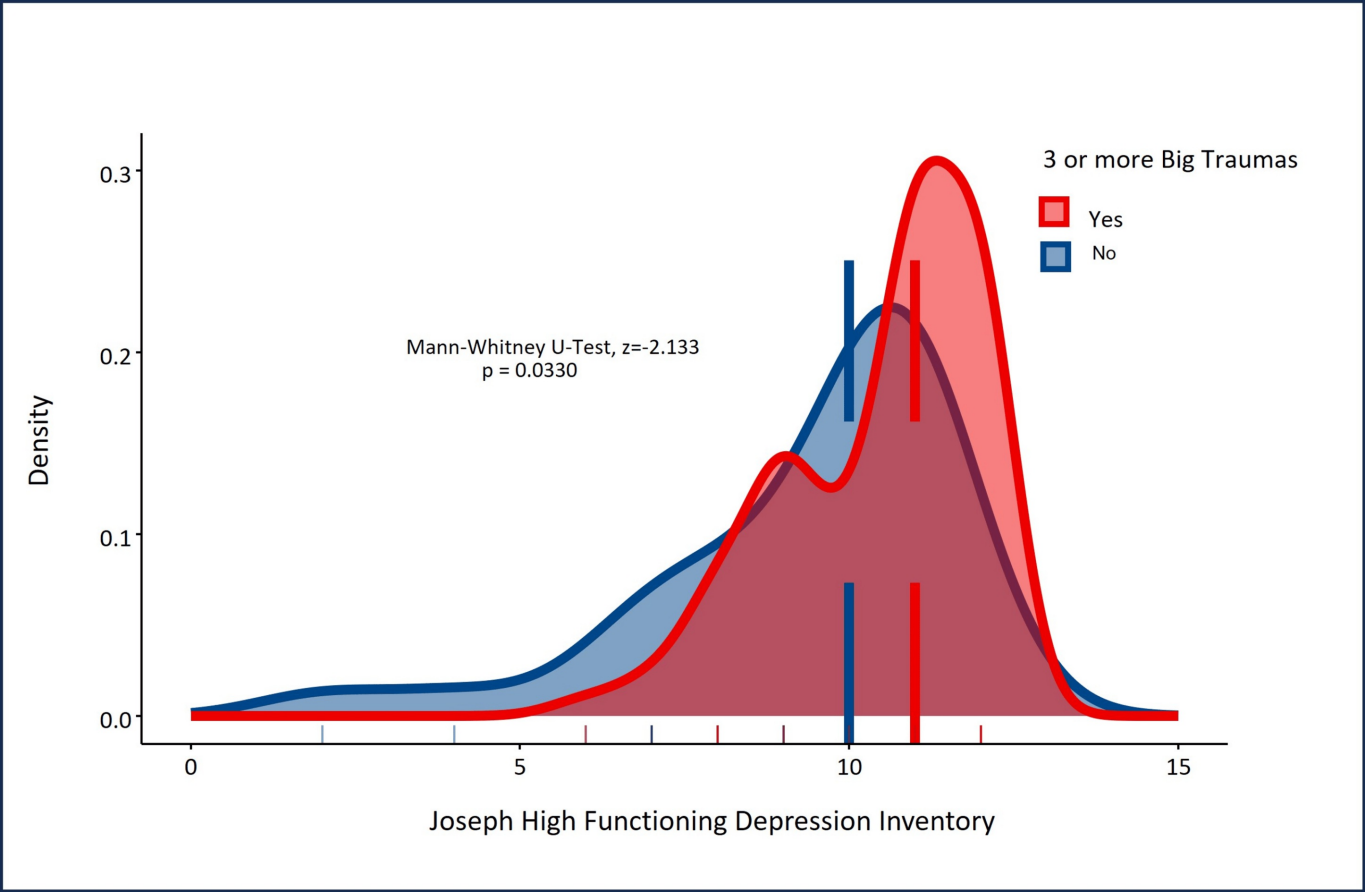
Density plot analyses showing higher HFD-I scores for participants with three or more big traumas (bigT3+) HFD-I: High-Functioning Depression Inventory

When participants were dichotomized according to the number of big traumas, mean HFD-I total scores were significantly higher in participants with three or more big traumas (bigT3+) compared to those who reported fewer than three big traumas (z=-2.133, p=0.033). Density plot analysis summarizing the divergence in HFD-I total scores between participants with bigT3+ and participants with two or fewer showed, as anticipated, that participants with bigT3 had significantly higher HFD-TI total scores than participants who reported fewer numbers of big traumas (z=-6.149, p<0.0001, Figure 6).

There was also a significant association between education level and bigT3+ (χ^2^=9.5751, df=3, p=0.023). Moreover, there was a statistically significant decrease (linear trend) in the frequency of bigT3+ as the educational level increased (Table 3). As shown in Table 3, 100% (n=8) of the high school graduate participants who were interviewed reported bigT3+, followed by 83.3% (n=20) of participants with some college, then 68.5% (n=37) of the college graduate participants, and 58.6% (n=17) of the graduate degree participants (z=3.052 p=0.002). This linear trend suggests that as education level increases, the number of big traumas decreases.

**Table 3:** Frequency association of big trauma and education level

	Three or more big traumas		Statistics
Education	No	Yes	
High school graduate	0 (0%)	13 (100%)	Linear-by-linear association test, Z=3.052, p=0.002
Some college	4 (16.7%)	20 (83.3%)	
College graduate	17 (31.5%)	37 (68.5%)	
Graduate degree	12 (41.4%)	17 (58.6%)	

Gender identity was also significantly associated with bigT3+, with 66.2% (n=51) of the female participants reporting bigT3+, followed by 80% (n=28) of the male participants and 100% (n=8) of the non-binary participants.

Secondary analyses of participants’ caregiver roles demonstrated that the median HFD-AS scores and HFD-I scores were significantly different across caretaker groups, which were parent/caregiver of children, caregiver of parents/elderly relative (e.g., elderly grandparent, parent, etc.), or neither. Follow-up analyses showed a significant decreasing linear trend in HFD-AS scores (JT=-773, z=-2.495, p=0.0126) and HFD-I scores (JT=-726, z=-2.308, p=0.0210), with parent/caretakers of children having the highest AS and HFD-I scores, followed by the caregivers of parents, and then participants who were neither type of caregiver. Furthermore, post hoc pairwise comparisons for the AS and the HFD-I by caregiver groups showed that the mean HFD-AS scores were significantly higher in the parent/caregiver of children group (W=1119.5, p=0.02154) than the "neither" caregiver group. The mean HFD-I scores were also significantly higher for the parents/caregiver of children group (W=1153.5, p=0.01622) than the "neither" caregiver group. Furthermore, HFD-I scores and HFD-TI scores were significantly correlated in the parent/caregiver of children group (ρ=0.534, p=0.0104) and the caregiver of parents group (ρ=0.634, p=0.027). The number of big traumas was also significantly correlated with HFD-I scores in the parent/caregiver of children group (ρ=0.546, p=0.0086).

## Discussion

The current interview study focused on using clinician-administered psychometric tools, such as the semi-structured HFD Analysis Questionnaire, the HFD-I, the HFD-TI, and the Joseph HFD-AS, to assess demographics, lifestyle patterns, and risk factors associated with HFD, the presence of distinctive HFD characteristics, and the frequency of traumas for people with HFD. Gender identity was significantly associated with the number of big traumas experienced, including three or more (bigT3+). For the population of participants interviewed, 100% of the non-binary participants reported bigT3+, followed by 80% of the male participants and 66% of the female participants. This indicated an increased occurrence of big trauma for non-binary participants. However, the difference between the number of participants in each gender identity group and the small number of participants in each gender identity group who reported bigT3+ (female: n=51, male: n=28, and non-binary: n=8) limits the interpretation of these findings. Larger sample sizes are necessary to identify a stronger potential trend between gender identity and the occurrence of big trauma experiences.

There was also a correlation between symptoms of HFD, such as anhedonia, and marital status, as post hoc tests showed that the average HFD-AS score was higher in the married or partnered participants than in those who were single. As anticipated based on symptoms observed in clinical practice among patients with suspected HFD, the participants with higher HFD-AS total scores also had higher HFD-I total scores. These participants also experienced higher HFD-TI scores and bigT3+ rates. Furthermore, as the education level of the participants increased, the number of big traumas, including bigT3+, decreased. Although the percentage of reported bigT3+ appeared to decrease with increasing education level, the number of individuals in each group was different, making it hard to determine what the actual percentage of bigT3+ would be if each of the groups had the same number of participants. In addition, reported traumas reflect events that happened prior to obtaining any form of college education or degree. Thus, the traumas preceded educational achievement, limiting the interpretation of educational level impacting the frequency of bigT3+.

Instead, upper-level educational attainment may be attributed to psychological resilience to the potential long-term impacts of big traumas [6], as in this population, most of the HFD participants with bigT3+ were still able to obtain some form of higher-level education. This indicates that education level may help build resilience to the impact of past trauma, whereas education level cannot decrease the actual occurrence of bigT3+. Moreover, the educational attainment of a higher-level degree appears to reflect resilience, which can be defined as people’s active attempts through multisystemic dynamic processes to protect themselves from potentially destabilizing conditions that can be induced by recalling actual events or thoughts of past traumatic experiences [6,22-24]. For example, a person who experienced a big trauma during childhood (e.g., witnessed a stabbing), a big trauma during adulthood (e.g., experienced sexual assault), and a mass big trauma (e.g., survived a massive earthquake) who went on to receive a graduate degree could not stop these events from happening but remained resilient and obtained the higher-level degree despite reporting bigT3+. Another possible explanation involves sublimation, as a high-functioning individual may channel the unacceptable urge to quit college, graduate school, or medical school into productive outlets that lead to the attainment of an upper-level degree [8]. However, such individuals may not realize or acknowledge the onset of HFD symptoms, such as increased levels of anhedonia, sadness, distress, poor concentration, or lack of emotion. Over time, the ability to employ psychological resilience or sublimation may weaken, causing even the high-functioning individual with an upper-level degree to begin to experience the adverse effects of untreated HFD. These findings warrant more appropriate assessment tools, such as the clinician-administered inventories used in this study, along with effective diagnostic and treatment approaches to help prevent long-term adverse events or the development of MDD.

Secondary analyses of the participants’ caretaker roles demonstrated that the median HFD-AS scores and HFD-I scores were significantly different across caregiver groups. In particular, the participants who were parents/caretakers of children had the highest AS and HFD-I scores, followed by the caregivers of parents or relatives. Parents or caregivers of children also had higher reports of bigT3+. As anticipated, these findings indicate that HFD caregivers experience higher levels of anhedonia that they have made attempts to cope with, which may cause them to overlook this symptom of depression. However, overlooking this symptom or being among the population of HFD individuals who are underdiagnosed and undertreated may put them at a greater risk for experiencing symptoms of MDD.

The US Surgeon General recently released a new public health warning, which states that most parents are facing what appears to be more stress than at any other time in history [25]. The report explains that parents often rate their level of stress as an 8 or higher on a 10-point scale and that most report feeling so stressed that they feel numb [25]. Parents who continue to provide care for their family, attain upper-level degrees, or execute demanding work tasks are displaying HFD that needs to be addressed.

It is theorized that demanding lifestyles (e.g., caregiving, being a physician) or intense trauma trigger individuals with HFD to engage in distracting behaviors, psychological resilience, or sublimation as a way to focus their skills, resources, energy, and time on areas other than their personal needs or past trauma [26,27]. However, in doing so, they may develop a lowered ability to acknowledge negative emotions, such as sadness or joy. This may be one of the reasons why anhedonia is a prominent mood state in these individuals. It is also proposed that if the symptoms persist, they may lead to adverse outcomes similar to typical depression. Furthermore, it is possible that individuals with HFD may develop typical depression or co-morbid substance abuse [27].

Another factor that may contribute to the underdiagnosis and undertreatment of HFD is that there is likely a high rate of HFD among medical professionals - a contributing factor that may be delaying the proper identification or pathologization of HFD. Indeed, if there is a high rate of a specific condition (e.g., HFD) among individuals within the medical community who help define conditions that are recognized by the DSM-5, then it is unlikely that these individuals will recognize their own symptoms of HFD as being an abnormal state or a condition that warrants treatment.

To promote a shift in mental health care for people experiencing HFD, we created constructed psychometric inventories to help elucidate the signs, symptoms, risk factors, and possible mechanisms for HFD. The findings identified symptoms and characteristics that appear to be specific to this population, thus offering a definition of what an individual with HFD is experiencing. We anticipate that these individuals are likely at risk for lifetime major depressive episodes, and the associations that were observed between gender, marital status, education level, and frequency of bigT3+ and the presence of HFD necessitate effective treatment approaches for this population.

The findings of this study have to be seen in light of some limitations. The first involves the limited access to validated questionnaires that measure HFD, as the instruments that are currently available (e.g., SHAPS, ACES, and the Philadelphia Expanded ACE Survey) are antiquated, dated, and not applicable to the targeted population. Thus, relevant questionnaires were designed based on previously developed instruments to evaluate the presence of HFD in individuals identified as high-functioning. The use of questionnaires that rely on self-reported data may have also introduced potential biases in the responses. Furthermore, the relatively small sample size for this study limits the generalizability of the current findings to the wider population. Due to the time limitations of the study, a more comprehensive analysis of certain variables (e.g., gender, race) was not possible, and finally, limited access to existing literature related to HFD may have restricted the scope of the analysis.

Future studies with larger sample sizes are warranted to further elucidate the impact of HFD on risk factors, such as the levels of daily functioning, anhedonia, sadness, and other prominent symptoms that may predict the occurrence of major depression. Additional research can also improve the understanding of the multisystemic processes of coping, psychological resilience, and sublimation in the educational attainment of higher-level degrees for some individuals with HFD, along with a clearer understanding of potential gender identity associations.

## Conclusions

This study aimed to identify individuals with HFD, explore their depression symptoms, and offer effective coping strategies. To date, there has not been a study on HFD because this term only emerged recently in popular discourse, and this health issue is not yet recognized in the DSM-5, although many people identify with its symptoms. However, a growing awareness and openness about discussing mental health issues may lead to more people seeking diagnosis and treatment for HFD. If increased awareness of how HFD presents in patients becomes recognized by the medical community, standardized diagnostic and treatment procedures may be incorporated into conventional interventions to help prevent HFD from progressing into more severe forms of depression.

The findings from this novel HFD interview study offer possible mechanisms, answers, and a deeper understanding of the symptoms people describe when they use the term "high-functioning depression" to describe their experiences. Currently, constraints in mental health care have made the prevention and treatment of HFD difficult. Many mental health issues and disorders do not receive a medical code or DSM-5 recognition until they cause significant distress or impairment. We argue that people with HFD are likely at high risk for developing impairment if their symptoms, such as anhedonia and increased levels of trauma, continue to go untreated and unaddressed. By advocating for a shift in focus to individuals with HFD, we may be able to prevent chronic mental health issues and lower the risk of HFD progressing to MDD.

## Appendices

Appendix A

Semi-structured HFD Analysis Questionnaire

Demographics:

**Age:  **  

       Black or African American                                                                                Hispanic

       White                                                                                                                  Non-Hispanic

       American Indian or Alaska Native

       Asian

       Native Hawaiian or Other Pacific Islander

       Other:                                             (if Other, please explain.)

Gender Identity:

       Male

       Female

       Non-binary

       Other:                                             (if Other, please explain.)

Highest Education Level:

A)  Lower than High School

B)  High School Graduate

C)  Some College

D)  College Graduate

E)  Graduate Degree

Occupation:

A)  Professional

B)  Student,

C)  Entrepreneur

D)  Domestic Worker

E Other                                             

Caretakers:

      Parent/Caretaker of children

      Caretaker of Parents/Elderly Relative (i.e. elderly parent, grandparents, etc.)

      Neither

Marital Status:

      Single

      Married/Partnered

      Divorced/Separated/Widowed

High Function Depression Analysis:

**Treatment History: **Have you ever had treatment with a mental health professional for psychological or emotional support?

Yes or No

If ‘Yes’:

1.       What was the diagnosis?                                                                                                   

2.       What type of treatment?                                                                                                   

Current Level of Functioning during HFD episode:

A)  Low functioning,

B)  Baseline (Normal)

C)  High Functioning

D)  Very high functioning

Level of Sadness/Depressed Mood during HFD episode

A.     None

B.     Mild

C.     Moderate

D.     Severe

E.      Extreme

Current Level of Distress during HFD episode

A.     None

B.     Mild

C.     Moderate

D.     Severe

E.     Extreme

Please note that this interview is confidential and is not being recorded nor is it to be recorded or reposted, do you agree?   

Appendix B

High-Functioning Depression Trauma Inventory

**Table 4:** HFD-TI There are 4 stages of trauma that we will discuss: Childhood, Adult, Intergenerational, and Mass. We will ask questions in each of these four stages. Please answer "Yes" or "No." Circle Yes or No HFD-TI: High-Functioning Depression Trauma Inventory; SARS: severe acute respiratory syndrome

Childhood trauma		
1. Childhood: Did you feel unsafe in your neighborhood growing up?	Yes	No
2. Childhood: Were you bullied by a peer prior to age 18?	Yes	No
3. Childhood: Have you seen anyone being beaten, stabbed, or shot in real life?	Yes	No
4. Childhood: Did your caretakers fail to make you feel important or special?	Yes	No
5. Childhood: Did your family cut sizes of meals or skip meals because there was not enough money in the budget of food for a significant period of time?	Yes	No
6. Childhood: Did you live with anyone who was depressed or mentally ill?	Yes	No
7. Childhood: Did you live with someone who was suicidal?	Yes	No
8. Childhood: Did you live with anyone who was problem drinker or alcoholic?	Yes	No
9. Childhood: Did you live with anyone who used illegal street drugs or who abused prescription medications?	Yes	No
10. Childhood: Did you live with anyone who served time or was sentenced to serve time in a prison, jail, or other correctional facility?	Yes	No
11. Childhood: Were you ever in foster care?	Yes	No
12. Childhood: Did you ever see or hear a parent, stepparent or another adult who was helping to raise you being yelled at, screamed at, sworn at, insulted or humiliated?	Yes	No
13. Childhood: Did you ever see or hear in your home a parent, stepparent or another adult who was helping raise you being slapped, kicked, punched, or beaten up?	Yes	No
14. Childhood: Did you ever see or hear a parent, stepparent or another adult who was helping to raise you being hit or cut with an object, such as a stick or cane, bottle, club, knife, or gun?	Yes	No
15. Childhood: Did you ever have a parent, stepparent or another adult living in the home swear at you, insult you or put you down?	Yes	No
16. Childhood: Did you ever have a parent, stepparent or another adult living in the home push, grab, shove or slap you?	Yes	No
17. Childhood: Did you ever have a parent, stepparent or another adult living in the home hit you so hard you had marks or were injured?	Yes	No
18. Childhood: Did you ever have a parent, stepparent or another adult living in the home act in a way that made you afraid that you would be physically hurt?	Yes	No
19. Childhood: did an adult or older relative, family friend or stranger who was at least five years older than yourself ever touch or fondle you in a sexual way or have you touch their body in a sexual way?	Yes	No
20. Childhood: Did an adult or older relative, family friend or stranger who was at least five years older than yourself ever attempt to have or have any type of sexual intercourse, oral, anal, or vaginal with you?	Yes	No
21. Childhood: Were your parents ever separated or divorced?	Yes	No
22. Childhood: Were you diagnosed with a serious medical condition or life-threatening injury?	Yes	No
23. Childhood: Did you experience significant discrimination because of your sexual identity, gender identity or gender expression? If sexual identity, which sexual identity do you identify with?	Yes	No
Adulthood trauma		
24. Adulthood: Have you ever worked in an unsupportive or toxic workplace for a significant period of time?	Yes	No
25. Adulthood: Were you in a relationship for a significant length of time with an intimate partner who was depressed or mentally ill?	Yes	No
26. Adulthood: Were you in a relationship for a significant length of time with an intimate partner who was suicidal?	Yes	No
27. Adulthood: Were you in a relationship for a significant length of time with an intimate partner who was a problem drinker or alcoholic?	Yes	No
28. Adulthood: Were you in a relationship for a significant length of time with an intimate partner who used illegal street drugs or who abused prescription medications?	Yes	No
29. Adulthood: Were you in a relationship for a significant length of time with an intimate partner who served time or was sentenced to serve time in a prison, jail, or other correctional facility?	Yes	No
30. Adulthood: Were you in a relationship for a significant length of time with an intimate partner where they swore at you, insulted you or put you down?	Yes	No
31. Adulthood: Were you in a relationship for a significant length of time with an intimate partner where you were pushed, grabbed, shoved or slapped?	Yes	No
32. Adulthood: Were you in a relationship for a significant length of time with an intimate partner where you were hit so hard you had marks or were injured?	Yes	No
33. Adulthood: Were you in a relationship for a significant length of time with an intimate partner where you were afraid that you would be physically hurt?	Yes	No
34. Adulthood: Were you ever sexually assaulted as an adult?	Yes	No
35. Adulthood: Did you ever go through a separation or divorce from your spouse?	Yes	No
36. Adulthood: Did you ever have a life-threatening injury?	Yes	No
37. Adulthood: Have you ever experienced significant debt, such as bankruptcy, foreclosure, reliance on government assistance?	Yes	No
38. Adulthood: Have you worked in a career or job where there is violence or exposure to death.	Yes	No
39. Adulthood: Have you experience significant discrimination because of your sexual identity, gender identity or gender expression.	Yes	No
Intergenerational trauma		
40. Intergenerational: In childhood or adulthood, did you often feel that you were treated badly or unfairly because of your race or ethnicity?	Yes	No
41. Intergenerational: Were you, your parents or grandparents survivors of genocide?	Yes	No
42. Intergenerational: Were you, your parents or grandparents refugees?	Yes	No
Mass trauma		
43. Mass: Have you directly experienced war?	Yes	No
44. Mass: Have you directly survived a major fatal disaster such as flood, fire, earthquake, etc.	Yes	No
45. Mass: Were you significantly impacted by a major pandemic such as, COVID-19, SARS?	Yes	No
46. Mass: Were you a survivor of a mass shooting?	Yes	No

Appendix C

The Joseph High-Functioning Depression Inventory (Clinician-Administered Scale)

The following questions must be answered during the subject’s current high-functioning depression episode. By definition, functioning is not impaired, and they are functioning at high levels. The clinician will elicit information about the subject’s age, education level, occupation, and current functioning level prior to administering the rating scale.

Current Level of Functioning during HFD episode

A)     Low functioning,

B)     Baseline (Normal)

C)     High Functioning

D)     Very high functioning

Level of Sadness/Depressed Mood during HFD episode

A)     None

B)     Mild

C)     Moderate

D)     Severe

E)     Extreme

Current Level of Distress during HFD episode

A)     None

B)     Mild

C)     Moderate

D)     Severe

E)     Extreme

1. During the majority of your HFD period, did you experience emotions less intensely? For example feeling numb or empty

2. During the majority of your HFD period, did you experience sleep changes (i.e. oversleeping or under sleeping)

3. During the majority of your HFD period, did you experience appetite changes (such as eating too much or too little)

4. During the majority of your HFD period, did you have feelings of not being good enough or feelings of inadequacy or guilt?

5. During the majority of your HFD period, did you ever have low energy or experience burn out or exhaustion?

6. During the majority of your HFD period, did you have feelings of hopelessness?

7. During the majority of your HFD period, did you have feelings that life is pointless, increased thoughts of death or suicide?

8. During the majority of your HFD period, did you experience poor concentration or difficulty performing tasks?

9. During the majority of your HFD period, did you feel restless or uneasiness or feel stuck?

10. During the majority of your HFD period did you feel sluggish, or slow?

11.  During the majority of your HFD period, did you feel that you no longer enjoy the things you used to or there is a diminished interest in things that used to bring you pleasure?

12. During the majority of your HFD period did you not ask for help because you did not want to burden others.

Appendix D

Joseph HFD Anhedonia Scale

**Table 5:** Joseph HFD-AS In your current high-functioning depression state, state if you "agree" or "disagree" with the following statements (circle response). HFD-AS: High-Functioning Depression Anhedonia Scale

Statements		
1. I often make “delayed future happiness statements”, such as “When I get this job, then I will finally be happy” or “When I fall in love one day, then I will finally be happy.	Agree	Disagree
2. I find it hard to enjoy resting or taking breaks because I feel restless and empty when I am not busy.	Agree	Disagree
3. I rarely take the time to savor my meals	Agree	Disagree
4. I rarely enjoy reading for leisure	Agree	Disagree
5. When I take naps, I don’t wake feeling refreshed.	Agree	Disagree
6. When I watch TV or movies (streaming included), I’m not engaged in the program	Agree	Disagree
7. When I make an effort to dress up, I rarely enjoy the experience.	Agree	Disagree
8. When others compliment me, I have a hard time enjoying praise.	Agree	Disagree
9. I have a difficult time enjoying social interactions with friends and/or family members	Agree	Disagree
10. It is hard to enjoy vacation and/or holidays	Agree	Disagree
11. I find it difficult to enjoy my favorite hobbies or activities.	Agree	Disagree
12. I find it hard to enjoy listening to music	Agree	Disagree
13. I have a difficult time enjoying physical intimacy or sexual behavior	Agree	Disagree
14. I rarely enjoy self-care activities (such as a warm bath, massage, mani-pedi)	Agree	Disagree
15. I rarely enjoy simple pleasures such as a nice cup of coffee, tasting my favorite beverage, smelling sweet scents	Agree	Disagree
16. I rarely enjoy scenic views or beautiful art	Agree	Disagree
17. I have a hard time enjoying the work that I produce because I often feel it is not good enough; I could have done better.	Agree	Disagree
18. I rarely enjoy simple pleasures such as a nice cup of coffee, tasting my favorite beverage, smelling sweet scents	Agree	Disagree
